# Development a new chewing problem directory and its validation for Korean elders

**DOI:** 10.1186/s12903-022-02290-3

**Published:** 2022-06-25

**Authors:** Huong Vu, Jong-Koo Lee, Hyun-Duck Kim

**Affiliations:** 1 Department of Preventive and Social Dentistry, School of Dentistry, Seoul National University, 101 Daehak-Ro, Jongno-Gu, Seoul, 03080 Republic of Korea; 2 Department of Family Medicine, College of Medicine, Seoul National University, Seoul, Korea; 3 Dental Research Institute, Seoul National University, Seoul, Korea

**Keywords:** Chewing problem, Oral health indicator, Questionnaire, Natural and rehabilitated teeth

## Abstract

**Objectives:**

This study aimed to develop a new chewing problem directory (CPD) and validate it with oral health indicators such as total occlusion force, number of natural and rehabilitated teeth (NRT), NRT posterior, natural teeth, natural teeth posterior, and dental status among Korean elders.

**Background:**

Chewing problem is the main oral health problem in elders. However, there has been no validated tool using both subjective and objective assessment of chewing problem.

**Subjects and methods:**

A total of 537 participants aged 65 years or more were randomly assigned into 2 subsamples: developing sample (n = 260) for developing and internally validating the new CPD as the 1st stage and confirmation sample (n = 277) for confirming validation of CPD as the 2nd stage. CPD was developed using three subjective questionnaires (general eating, chewing nuts, and chewing meat problem) and objective NRT. Periodontitis, age, sex, education, smoking, alcohol drinking, metabolic syndrome, and frailty were considered as confounders. Following the development of CPD, CPD was validated using multiple multivariable logistic regression after controlling for confounders in confirmation sample and total sample.

**Results:**

The Cronbach’s alpha value for three subjective questionnaires of CPD was 0.87. Among oral health indicators, NRT (0–28) showed the highest impact association with subjective chewing problem score (partial r = − 0.276). The chewing problem from the new CPD was associated with all items of oral health indicators. The prevalence of chewing problems by CPD was 57.7% in developing sample. Elders with NRT ≤ 24, compared with those with NRT ≥ 25, showed the highest impact on chewing problems by new CPD (Odds Ratio = 7.3 in the confirmation sample and 5.04 in the total sample, *p* < 0.05) among oral health indicators.

**Conclusion:**

This new CPD was developed as a valid tool to evaluate the chewing problem for Korean elders in dental clinics and community-based settings.

## Introduction

Population aging in Korea has been fast-growing to a super-aged society in 2026 [1], accompanying many oral health problems, including tooth loss and chewing difficulties [2]. The chewing problem has been highlighted as major oral health in Korean elders, which has been associated with oral health problems encompassing occlusion force, the number of teeth rehabilitation, and systemic health such as dementia, cognitive impairment, and frailty [3–8]. In addition, impaired chewing ability could also raise undernutrition [4]. Thus, chewing ability is a critical factor in maintaining health and oral health for the aging population.

Traditionally, subjective questionnaires have been used to evaluate chewing ability or chewing difficulty in community studies [7, 9–12]. Some questionnaires considered the ability to eat different types of food classified from soft to hard [3, 4], which may be confused to respond correctly for the elders.

Recently, objective tests, including sieving comminuted food, artificial food, and color-changing chewing gums, have been used to evaluate chewing ability in the community [13, 14]. However, the results of these objective tests were not consistent with the subjective assessment [15]. Hence, it was suggested that chewing performance should be assessed using both objective and subjective methods [15]. Although a previous study used subjective and objective methods to evaluate the chewing problem [7], it was not validated. Therefore, there is a need to develop a new chewing problem directory (CPD) and validate it with oral health indicators.

Hence, this study aimed to develop the new CPD using subjective questionnaires and objective oral health indicators using a developing sample, and validate it using content, construct in the developing sample, and criterion validation in the developing and confirmation sample. For criterion validation, we made a hypothesis that chewing problem was associated with oral health indicators encompassing total occlusion force (TOF), number of natural and rehabilitated teeth (NRT), number of natural teeth (NT), and dental status (DS) such as dentate, partial denture, and complete denture after controlling for age, sex, education level, smoking, drinking, metabolic syndrome, and frailty among Korean elders.

## Materials and methods

### Ethical considerations and study design

This cross-sectional study was approved by the Institutional Review Board for Human Subjects at the Seoul National University School of Dentistry (approval number: S-020190017) and the Seoul National University Hospital Biomedical Research Institute (IRB approval number: C-1803-117-932). This study for the new CPD included two stages: first stage for development and internal validation, and second stage for validation as a confirmation. All participants provided written informed consent to their records. All participants were recruited as the baseline (2018–2019) of the community health education cohort. This is a collaboration project to assess medical and dental health in elderly in Songbuk-Gu, Seoul, Korea. Participants joined the survey voluntarily after several weeks of the advertising period. Systemic health status and oral health status were assessed by trained medical and dental health professionals in the project who received calibration training beforehand.

### Study population

Songbuk-Gu with 0.44 million residents in Seoul metropolitan city with 9.8 million residents in 25 Gus (city-level administrative division) was select as a pilot program area of the community health promotion program for Korean elders by the Korea Centers for Disease Control and Prevention (KCDC), because Songbuk-Gu was a representative cluster of elders in Korea [16]. The proportion of the population aged 65 and over was 16.5%, which was almost the same as the average of 16.0% in Seoul and in Korea [17]. The participants were randomly recruited in all 20 stratified Dongs (administrative sub-divisions) of Songbuk-Gu. They voluntarily registered to the survey after taking the information about the program from local health center personnel via phone call. On the day of the survey, the participants joined the survey at the local government health center for this study.

The inclusion criteria were five-fold: (1) elders aged 65 and above who lived in Songbuk-gu, (2) elders who do not live in nursing homes or clinics, (3) elders without critical diseases such as cancer and paralysis, (4) elders able to communicate and agree to follow the study procedures with written informed consent. (5) Participants with missing information were excluded. From a total of 73,158 elders aged 65 and above of Songbuk-gu, 743 participants were recruited in this study. Out of them, 206 participants with incomplete information were excluded. Finally, 537 participants were included in the final analysis. All participants were randomly assigned to two subsamples: the “developing sample” for the first stage and the “confirmation sample” for the second stage. In the first stage, the “developing sample”, which has 260 elders, was used to develop and internally validate the CPD. In the second stage, the “confirmation sample”, which included 277 elders, was used to confirm the validity of the new CPD.

### Development of chewing problem directory

**Fig. 1:**
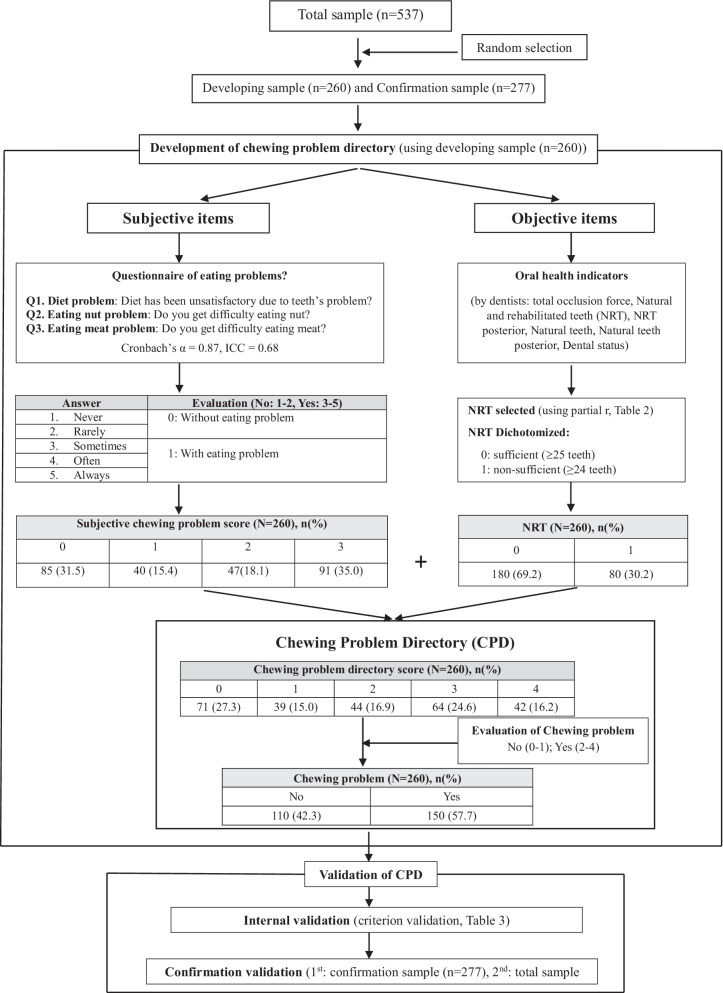
Flowchart for development of chewing problem directory and its validation

According to the guideline for developing new instrument approaches of Hulley [18] and COSMIN [19], we developed a new CPD and validated it. Participants were randomly assigned to either the developing sample group or the confirmation sample group using the random select case function of SPSS. A total of 260 participants were selected to develop and validate CPD, while data from the remaining 277 participants was used to validate it as a confirmation procedure. Finally, the CPD was validated again in the whole sample. (Fig. 1).

Subjective items of CPD included three check-up questionnaires (Fig. 1). The first question was about the general diet problem, “Has your diet been unsatisfactory due to teeth problems?” from the Oral Health Impact Profile (OHIP) inventory [20]. The second question was about the difficulty in chewing nuts (hard food), “Do you get any difficulty in chewing nuts?”[21, 22]. The final question was about the difficulty in chewing tough meat or rib (tough food) “Do you get any difficulty in chewing meat (tough meat or rib)?” [21] The response to three questionnaires was evaluated using the Visual Analog Scale with a five-level Likert scale (1: never, 2: rarely, 3: sometimes, 4: often, 5: always) [23]. Then, the response was dichotomized (0 = never or rarely, 1 = sometimes, often, always).

The objective item of the chewing problem was developed as the highest correlation coefficient among objective oral health indicators, according to the results of the linear regression model of oral health indicators for subjective chewing problem score (Table 2). Oral health indicators were measured by dentists through the oral exam. NRT was selected as the objective item. The cut-off value of NRT for subjective chewing problem was set at 25 (0 denotes NRT ≥ 25, and 1 denotes NRT ≤ 24) according to the results of the difference in NRT across the subjective chewing problem score. (Table 2).

The CPD score (ranging from 0 to 4) was calculated by summing up the dichotomized response about three subjective questionnaires and one objective NRT. For evaluating chewing problem, the CPD score of this new CPD was reclassified into two: no chewing problem (CPD score 0–1) and chewing problem (CPD score 2–4) (Fig. 1).

### Assessment of oral health indicators

Items of oral health indicators included total occlusal force (TOF) [24], number of natural and rehabilitated teeth (NRT) [25], number of natural teeth (NT) [26], and dental status (DS)[26] such as dentate, partial denture, and complete denture. The number of teeth and functioning teeth were also considered a critical determinant of chewing performance [9, 10, 22, 27]. Moreover, TOF, a quantitative measurement of chewing function, has been correlated with chewing function [6, 8, 28, 29]. Previous studies also show that DS could affect chewing performance [30]. We assessed DS as a categorical variable and TOF, NRT, NRT posterior, NT, and NT posterior as continuous variables.

DS included dentate, removable partial denture, and complete denture. Partial denture denotes having any removable partial denture without a complete denture. Complete denture denotes wearing at least one complete denture. During the oral examination, dentists counted NRT and NT using the naked eye under the blue light in the mobile dental unit chair. Pontic of fixed bridge and implants were considered as rehabilitated teeth [31]. Wisdom teeth were excluded from the analysis.

TOF as the maximal occlusal force was measured using pressure‐sensitive film sheets (Dental Prescale II 50H, GC Corp., Tokyo, Japan), a dedicated scanner (GT-X830, Epson, Tokyo, Japan), and analysis software (Bite Force Analyzer, GC Corp.). The maximal TOF was evaluated in Newton (N). Participants were instructed to bite the pressure-sensitive horseshoe-shaped film in the intercuspal position as strongly as possible in three seconds. [32] Denture wearers were recommended to keep their dentures in the mouth during the measurement. After calibration, the analysis of bite films was performed, and dentists carried out manual removal of artifacts according to the manufacturer’s guidelines. The limitation, validity, and reliability of Prescale II have been reported previously [32, 33]. Two dentists performed artifact erasing and measuring the TOF. For the reliability of TOF, 10% of the films were planned to retest. The interclass correlation coefficient between two dentists for 50 films was 0.97 and the intraclass correlation coefficient between two times tests of each dentist for 20 films was 0.96.

For sensitivity analysis for logistic regression models on dichotomized chewing problem from the new CPD, TOF, NRT, NRT posterior, NT, and NT posterior were dichotomized (sufficient versus non-sufficient) according to the difference in values across subjective chewing problem score: ≥ 350 N versus < 350 N for TOF, ≥ 25 versus ≤ 24 for NRT, ≥ 14 versus ≤ 13 for NRT posterior, ≥ 15 versus ≤ 14 for NT and ≥ 8 versus ≤ 7 for NT posterior. (Table 2).

### Assessment of confounders

For information regarding socio-demo-behavioral confounders, participants were interviewed face-to-face. Interviewers were recruited from the survey area and trained before the main survey using structured questionnaires. The social factor was the educational level. Demographic factors included age and sex. Alcohol drinking and smoking were considered as behavioral confounders.

Physicians performed physical examinations for a general health assessment. Blood samples were collected in the morning after 8 h of fasting, and all biochemical markers were analyzed on the same day. HDL cholesterol, triglyceride, and glycated hemoglobin (HbA1c) were measured (ADVIA1650 Automatic Analyzer, Bayer, Stillwater, MN). According to ATP III guideline [34], metabolic syndrome was diagnosed when having three or more factors among the following factors: (1) Obesity (body mass index (body kg/height m^2^) ≥ 25); (2) Total triglyceride ≥ 150 mg/dL; (3)HDL cholesterol: male < 40 mg/dL, female < 50 mg/dL or medication for dyslipidemia; (4) Hypertension: systolic blood pressure ≥ 130 mmHg or diastolic blood pressure ≥ 85 mmHg or medication for hypertension; (5) HbA_1_C ≥ 5.3% or medication for diabetes. For periodontal examination, clinical attachment loss was measured by dentists using UNC probe according to the guideline “Staging and grading of periodontitis: Framework and proposal of a new classification and case definition” [35]. Tooth loss due to periodontitis was determined using interviews by dentists. Established periodontitis was classified into two groups: No (healthy or stage I–II) and Yes (stage III–IV) [36].

Frailty was determined using the FRAIL scale [37], including five components: fatigue, resistance, ambulation, illness, and weight loss. FRAIL scale scores range from 0 to 5 (one point for each component; 0 = best to 5 = worst). Less than 3 points indicated no frailty, and 3 points or above was frailty.

### Statistical analysis

Cronbach’s alpha and inter-intraclass of correlation coefficient (ICC) were estimated to evaluate the internal consistency of the three subjective questionnaires in CPD.

The outcome was chewing problem (no versus yes). The main explanatory variables were oral health indicators: TOF, DS, NRT, NRT posterior, NT, and NT posterior. Periodontitis, age, sex, education, smoking, alcohol drinking, metabolic syndrome, and frailty were considered as confounders.

Differences in characteristics between positive and negative chewing problem were evaluated using bivariate analyses such as T‐test for continuous variables and chi‐square test for categorical variables. The characteristics of participants were described using frequency distributions for categorical variables and means with standard deviations for continuous variables. The analysis of variance (ANOVA) and covariance (ANCOVA), including the Bonferroni post-hoc multiple comparison test, was applied to compare the mean and the adjusted mean across the subjective questionnaires score (0–3), respectively.

Multiple multivariable linear regression analysis was applied to evaluate the association between oral health indicators and subjective chewing problem score (range 0 to 3) after controlling for confounders. The partial regression coefficient was estimated to compare the impact association of each oral health indicators on the subjective chewing problem score.

Multiple multivariable logistic regression analysis was applied to evaluate the association (odds ratio, [OR]) of categorized oral health indicators with chewing problem after controlling for confounders in confirmation sample and the total sample.

All analyses were performed using SPSS version 25.0 (SPSS, Inc., Chicago, IL). Statistical significance was set at *p* < 0.05.

## Results

### Developing CPD

There was no significant difference in demographic factors and oral health indicators between the developing and confirmation sample, except for the prevalence of alcohol drinking (Table 1). Internal consistency of the three subjective questionnaires in CPD was deemed very good (Cronbach’s alpha = 0.866, *p* < 0.001; ICC = 0.683, *p* < 0.001) (Fig. 1). The prevalence of the chewing problem in the developing sample and the total sample was 57.7% and 52.7%, respectively.

**Table 1:** Characteristics of participants

Variable	Developing sample (n = 260)	Confirmation sample (n = 277)	*p* value
Age, year	76.11** ± **5.01	76.50 ± 5.22	0.383
Sex			0.555
Male	84 (30.3)	85 (32.7)	
Female	193 (69.7)	175 (67.3)	
Education^a^			0.763
Middle school or less	214 (77.3)	198 (76.2)	
High school or more	63 (22.7)	62 (23.8)	
Smoking^b^			0.539
No	186 (67.1)	181 (69.6)	
Yes	91 (32.9)	79 (30.4)	
Alcohol drinking^c^			0.043
No	105 (37.9)	77 (29.6)	
Yes	172 (62.1)	183 (70.4)	
Periodontitis^d^			0.081
No	60 (21.7)	41 (15.8)	
Yes	217 (78.3)	219 (84.2)	
Metabolic syndrome^e^			0.308
No	125 (45.1)	106 (40.8)	
Yes	152 (54.9)	154 (59.2)	
Frailty, n (%)			0.223
No	228 (83.2)	224 (86.2)	
Yes	49 (17.7)	36 (13.8)	
Dental status			0.470
Dentate	162 (58.5)	160 (61.5)	
Denture	115 (41.5)	100 (38.5)	
TOF, Newton	364.67 ± 314.67	369.51 ± 313.02	0.862
NRT	25.42 ± 4.16	25.44 ± 3.91	0.952
NRT Posterior	13.78 ± 3.26	13.86 ± 3.08	0.764
NT	16.39 ± 8.51	16.57 ± 8.76	0.814
NT Posterior	8.23 ± 5.18	8.01 ± 5.07	0.618

Values denote as number (column percentage) for categorical variables and mean ± standard deviation (SD) for continuous variables

TOF, total occlusion force; NRT, natural/rehabilitated teeth number; NT, natural teeth number; Posterior, premolars and molars

*p* value was obtained from chi-square test for categorical variables and from T-test for continuous variables

^a^Education: "Middle school or less" refers to be educated until 9 years, the graduation of middle school, "High school or more" refers to be educated more than 9 years, entered into high school or more

^b^Smoking: “No” refers to never smoked, “Yes” refers to past and current smoker

^c^Alcohol drinking: “No” refers to drunken, “Yes” refers to past and current drinker

^d^Periodontitis: followed by guideline “Staging and grading of periodontitis: Framework and proposal of a new classification and case definition” (Tonetti et al., 2018) classified into two groups: No (healthy or stage I-II) and Yes (stage III-IV)

^e^Metabolic syndrome: “No” refers to two or fewer factors, “Yes” refers to three or more factors among five factors: Obesity (body mass index (body kg/ height m^2^) ≥ 25); Total triglyceride ≥ 150 mg/dL; HDL cholesterol: Male < 40 mg/dL, Female < 50 mg/dL or medication for dyslipidemia; Hypertension: systolic blood pressure ≥ 130 mmHg or diastolic blood pressure ≥ 85 mmHg or medication for hypertension; Glycated hemoglobin ≥ 5.3% or medication for diabetes

Oral health indicators encompassing TOF, NRT, NRT posterior, NT, and NT posterior across subjective chewing problem scores were significantly different in crude and adjusted values (Table 2). The subjective chewing problem, defined by subjective CPD score ≥ 2, showed lower values of TOF (< 350 N), NRT (≤ 24), NRT posterior (≤ 13), NT (≤ 14), and NT posterior (≤ 7) (*p* < 0.05). NRT showed the highest correlation coefficient (partial r = –0.276) with the subjective chewing problem score among oral health indicators in the multivariable linear regression after controlling for confounders. (Table 2) Therefore, NRT was selected as an objective assessment in CPD (Fig. 1).

**Table 2:** Oral health indicators across subjective chewing problem score (0–3) in developing sample (n = 260)

Variable	Subjective chewing problem score				Partial r	*p* value
	0 (n = 82)	1(n = 40)	2(n = 47)	3(n = 91)		
TOF, Newton						
Crude^1^	461.8 ± 394.1^a^	366.4 ± 281.4^a,b^	337.8 ± 261.9^a,b^	290.5 ± 247.5^b^		0.004
Adjusted^2^	444.7 ± 33.5^a^	365.7 ± 47.1 ^a,b^	351.5 ± 43.5^a,b^	299.3 ± 31.7^b^	− 0.202	0.023
NRT						
Crude^1^	27.3 ± 2.3^a^	26.7 ± 2.6^a^	25.6 ± 4.8^a,b^	24.4 ± 5.6^b^		< 0.001
Adjusted^2^	27.0 ± 0.4^a^	25.9 ± 0.6^a^	25.0 ± 0.5^a^	24.0 ± 0.4^b^	− 0.276	< 0.001
NRT posterior						
Crude^1^	15.4 ± 2.2^a^	14.7 ± 2.4^a^	14.1 ± 3.5^a^	13.8 ± 4.1^b^		< 0.001
Adjusted^2^	15.0 ± 0.3^a^	14.0 ± 0.5^a,b^	13.3 ± 0.4^b,c^	12.8 ± 0.3^c^	− 0.263	< 0.001
NT						
Crude^1^	19.8 ± 8.4^a^	17.3 ± 8.8^a,b^	15.3 ± 10.1^b^	15.4 ± 9.0^b^		0.006
Adjusted^2^	18.0 ± 0.6^a^	17.1 ± 0.8^a,b^	16.1 ± 0.7^a,b^	15.3 ± 0.5^b^	− 0.189	0.006
NT posterior						
Crude^1^	10.2 ± 5.5^a^	8.7 ± 5.4^a,b^	7.8 ± 6.2^a,b^	7.8 ± 5.4^b^		0.027
Adjusted^2^	9.0 ± 0.4^a^	8.6 ± 0.4^a,b^	8.4 ± 0.4^a,b^	7.8 ± 0.3^b^	− 0.157	0.085

TOF, total occlusion force; NRT, natural/rehabilitated teeth number; NT, natural teeth number; Posterior, premolars and molars; Partial r, correlation coefficient

Crude^1^: Mean ± Standard deviation; Adjusted.^2^: Mean ± Standard error

Superscript denotes the same subgroups by Bonferroni’s post-hoc multiple comparisons test

*p* value: obtained from ANOVA for crude values and ANCOVA for adjusted value after controlling for sex, age, alcohol drinking, smoking, education, periodontitis, metabolic syndrome and frailty

### First-stage criterion validation of CPD

In the developing sample, participants with chewing problem (CPD ≥ 2) showed lower TOF (in crude: 316.39 ± 258.65 N versus 430.82 ± 369.07 N; in adjusted: 320.09 ± 24.41 N versus 425.77 ± 28.64 N), NRT (in crude: 24.27 ± 4.94 versus 27.00 ± 1.84; in adjusted: 24.36 ± 0.30 versus 26.87 ± 0.35), NRT posterior (in crude: 12.85 ± 3.75 versus 15.03 ± 1.81; in adjusted: 12.93 ± 0.23 versus 14.93 ± 0.27), NT (in crude: 15.15 ± 8.71 versus 18.49 ± 8.50; in adjusted: 15.31 ± 0.46 versus 18.27 ± 0.54), and NT posterior (in crude: 7.47 ± 5.01 versus 9.29 ± 5.24; in adjusted: 7.55 ± 0.29 versus 9.19 ± 0.34) than those without chewing problem (*p* < 0.001). (Table 3) According to results of multiple multivariable linear regression analysis of oral health indicators for chewing problem, the impact of association with chewing problem was highest in NRT posterior (partial r = − 0.327), followed by NRT (partial r = − 0.32), NT (partial r = − 0.254), NT posterior (partial r = − 0.232), and TOF (partial r = − 0.176), in order.

**Table 3:** Oral health indicators according to chewing problem by chewing problem directory in developing sample: internal criterion validation (n = 260)

Variable	Chewing problem		Partial r	*p* value
	No (n = 110)	Yes (n = 150)		
TOF, Newton				
Crude^1^	430.82 ± 369.07	316.39 ± 258.65		0.004
Adjusted^2^	425.77 ± 28.64	320.09 ± 24.41	− 0.176	0.006
NRT				
Crude^1^	27.00 ± 1.84	24.27 ± 4.94		< 0.001
Adjusted^2^	26.87 ± 0.35	24.36 ± 0.30	− 0.320	< 0.001
NRT posterior				
Crude^1^	15.03 ± 1.81	12.85 ± 3.75		< 0.001
Adjusted^2^	14.93 ± 0.27	12.93 ± 0.23	− 0.327	< 0.001
NT				
Crude^1^	18.49 ± 8.50	15.15 ± 8.71		0.002
Adjusted^2^	18.27 ± 0.54	15.31 ± 0.46	− 0.254	< 0.001
NT posterior				
Crude^1^	9.29 ± 5.24	7.47 ± 5.01		0.005
Adjusted^2^	9.19 ± 0.34	7.55 ± 0.29	− 0.232	< 0.001

TOF, total occlusion force; NRT, natural/rehabilitated teeth number; NT, natural teeth number; Posterior, premolars and molars; Partial r, correlation coefficient

Crude^1^: Mean ± Standard deviation; Adjusted.^2^: Mean ± Standard error

Superscript denotes the same subgroups by Bonferroni’s post-hoc multiple comparisons test

*p* value: obtained from T-test for crude values and ANCOVA for adjusted value after controlling for sex, age, alcohol drinking, smoking, education, periodontitis, metabolic syndrome and frailty

### Second-stage confirmation criterion validation of CPD

According to the results of multiple multivariable logistic regression analysis, chewing problem (no versus yes) from the new CPD was independently associated with all items of oral health indicators in both the confirmation sample and total sample (Table 4). In confirmation sample, among oral health indicators, elders with NRT ≤ 24 showed the highest odds of chewing problem by 7 times (OR = 7.30; CI 3.55–14.99) followed by NT ≤ 14 by 2.5 times (OR = 2.48; CI 1.45–4.27), NT posterior ≤ 7 by 2.4 times (OR = 2.45; CI 1.45–4.15), and denture wearer by 2.3 times (OR = 2.28, CI 1.35–3.83). TOF < 350 N and NRT posterior ≤ 7 showed the odds of chewing problem by 2.1 times (OR = 2.10; CI 1.26–3.5 and OR = 2.12; OR = 1.26–3.57, respectively).

**Table 4:** Association of oral health indicators with chewing problem (no versus yes) by chewing problem directory in confirmation sample

Variables	OR (95% confidence interval)			
	Confirmation sample (n = 277)		Total sample (n = 537)	
	Crude	Adjusted	Crude	Adjusted
Dental status				
Dentate	1	1	1	1
Denture	**2.30 (1.41–3.74)**	**2.28 (1.35–3.83)**	**1.54 (1.08–2.18)**	**1.46 (1.01–2.12)**
TOF, Newton				
≥ 350	1	1	1	1
< 350	**2.34 (1.44–3.81)**	**2.10 (1.26–3.50)**	**1.98 (1.40–2.81)**	**1.86 (1.30–2.68)**
NTR				
≥ 25	1	1	1	1
≤ 24	**7.67 (3.80–15.45)**	**7.30 (3.55–14.99)**	**5.12 (3.33–7.88)**	**5.04 (3.24–7.83)**
NTR posterior				
≥ 14	1	1	1	1
≤ 13	**2.25 (1.37–3.70)**	**2.12 (1.26–3.57)**	**3.14 (2.17–4.55)**	**3.06 (2.09–4.48)**
NT				
≥ 15	1	1	1	1
≤ 14	**2.51 (1.54–4.10)**	**2.48 (1.45–4.27)**	**2.07 (1.46–2.95)**	**1.99 (1.36–2.91)**
NT posterior				
≥ 8	1	1	1	1
≤ 7	**2.45 (1.51–3.98)**	**2.45 (1.45–4.15)**	**1.71 (1.21–2.41)**	**1.64 (1.36–2.37)**

Outcome: chewing problem (no versus yes)

Odds ratio: obtained by multiple multivariable logistic regression adjusted for age, sex, education, periodontitis, smoking, alcohol drinking, metabolic syndrome and frailty

TOF, total occlusion force; NTR, natural/rehabilitated teeth number; NT, natural teeth number; Posterior, premolars and molars

Bold font indicates statistical significance

In the total sample, among oral health indicators, NRT ≤ 24 still demonstrated the highest odds of the chewing problem by five times, followed by NRT posterior by three times and NT ≤ 14 by two times. Also, TOF < 350, NT posterior ≤ 7, or denture increased the risk of chewing problem in the elderly by 1.9 times, 1.6 times, and 1.5 times, respectively. (Table 4).

## Discussion

The chewing problem is common in older adults aged 65 or more [3, 7]. In Korea, chewing problem has been a major national oral health indicator for elders aged 65 or more. The chewing problem is associated with many factors including oral and systemic diseases and aging. Hence, more solid tool for evaluating chewing problem correctly is needed for elders.

According to our results, the aims of the study were met. This new CPD was developed and thoroughly validated through content, construct, and criterion validation at the first stage of this study. At the second stage, the new CPD was also validated as a confirmation using another sub-sample. Thus, this new CPD could be a solid and practical tool to assess the chewing problem for elders.

This study has five major strengths. Firstly, this new CPD was validated through three steps: content, construct, and criterion validation, according to the guideline of Hulley [18]. Secondly, the CPD applied both subjective and objective assessments, making the evaluation more reliable than when used only a single method. Thirdly, the new CPD was validated using TOF as an oral health indicator by the commercial Dental Presale II system. Also, the association was adjusted for well-known potential confounders, including sociodemographic, behavioral, oral, and systemic health factors. Last but not least, this CPD was confirmed in a similar sub-population.

Some limitations and future studies are as follows. Firstly, due to the cross‐sectional study design, predictive validity could not be evaluated. Secondly, the variation of occlusal force measurement was considerable among participants with similar oral conditions. Finally, we did not consider the salivary flow rate [38], nutritional status [4], and cognitive impairment [39], which could also affect the chewing problem. A longitudinal study showed that the number of residual teeth strongly affected long-term declines in chewing function [6]. Hence, future longitudinal studies will clarify the predictive validity for this new CPD and the causality of functioning teeth on chewing problem diagnosed by this new CPD. Moreover, this CPD could be applied to other ethnic population in the world and 40th or 50th age groups with low prevalence of chewing problem in the future.

Chewing is a complex process in which foods are broken down into small fragments by grinding and shearing and mixed with saliva before swallowing. Chewing performance has been evaluated objectively using color-changed chewing gum [3, 7], gummy jelly [40], or the sieving method [41]. Since the chewing problem was speculated to include subjective sensation[4], it can also be assessed using various questionnaires. Moreover, the self-reported chewing problems were more reliable than the quality of the dentition itself as an indicator of altered nutritional status [42]. However, questionnaires on chewing problems could not cover the specific chewing performance. [3, 4, 7, 9, 10, 43–46]

This new CPD included subjective and objective approaches to evaluating chewing problem. Some studies showed that the subjective and objective chewing function were different [44, 47]; while one study showed that subjective and objective chewing functions were similar in the same population [10]. Thus, it is persistent in using the subjective questionnaires and objective oral health indicators simultaneously. Since NRT was the highest impact of association with subjective chewing problem score by the multivariable linear regression model, the objective evaluation of NRT was added to develop the new CPD. Thus, this new CPD using three questionnaires and NRT assessment showed content validity by the previous studies[20, 21]. To do a criterion validation for CPD, it is reasonable to compare it with conventional indicators such as NRT and TOF, because lower values of OH indicators have been associated with the chewing problem strongly [6, 8–10, 22, 27–30]. The prevalence of chewing problem was 57.7% in the developing sample and 52.7% in total sample, which were substantially acceptable compared to the prevalence of chewing problem for the questionnaire (45.3–51.0% for a single item, 53.1% for three items in developing sample).

This new CPD was associated with TOF. This result was consistent with previous studies showing that less occlusal force was related to chewing problem [6, 28, 48]. The occlusal force was made by the chewing muscle, whose strength transmits via teeth to food. Thus, a decrease in TOF could impair the ability to chew hard or chewy foods such as vegetables, fish, or shellfish [49]. Additionally, the CPD score was associated with NRT and NT. The strong association between the number of functional teeth and chewing ability has been revealed in many studies [6, 48, 50]. Our study confirmed the association between the number of natural and rehabilitated teeth and TOF with chewing problem after controlling for various confounders.

Impaired chewing function impacts nutritional intake, worsening general health, and quality of life in elders. Hence, uncovering the association between the chewing problem and oral health indicators contributes to preventive modalities for oral/general health and promotion of quality of life. The most important and influential methods for chewing function are protecting natural dentition and prosthetic replacement of missing teeth, because having more functional teeth could improve chewing ability [47]. Not only that, better adherence to a dietary pattern characterized by a high intake of green leafy vegetables and a low intake of rice showed a positive association with the number of remaining teeth [51]. Additionally, we should maintain sufficient saliva to make small portions of broken foods into a bolus, facilitating swallowing. Finally, chewing muscle training via chewing exercises could enhance occlusal force [52], and the application of chewing gum to elders could be an easy chewing exercise [53].

## Conclusion

This new chewing problem directory was developed and validated. This chewing problem directory could be a practical tool to evaluate the chewing problem for elders in dental clinics and community-based studies.

## Data Availability

The datasets used and/or analyzed during the current study are available from the corresponding author on reasonable request.
